# Faculty of Education Professors’ Perception about the Inclusion of University Students with Disabilities

**DOI:** 10.3390/ijerph182111667

**Published:** 2021-11-06

**Authors:** Rosa-Eva Valle-Flórez, Ana María de Caso Fuertes, Roberto Baelo, Sheila García-Martín

**Affiliations:** 1 Department of General and Specific Didactics and Educational Theory, University of León, 24071 León, Spain; rosa-eva.valle@unileon.es (R.-E.V.-F.); sgarcm@unileon.es (S.G.-M.); 2 Department of Psychology, Sociology and Philosophy, University of León, 24071 León, Spain; amcasf@unileon.es

**Keywords:** disability inclusion, higher education, special educational needs (SEN), universal design for learning (UDL), inclusive education, teacher training

## Abstract

The recognition of the right to the inclusion of people with disabilities on a global scale has led to progress in the planning and development of policies and programs among different areas. The present work addresses the accessibility barriers to university education as an opportunity for greater inclusion. Universities must facilitate all students’ physical, curricular, and relational access and comply with a “reasonable accommodation” policy to allow students with disabilities equal opportunities. The aim is to analyze the barriers that hinder educational inclusion. Four factors are explored: the students’ accessibility to facilities and resources; the teachers’ willingness to respond to students with disabilities and special educational needs (SEN); the teachers’ curricular adjustments to meet those needs; the students’ interactions with their peers and professors. We worked with a sample of 201 university professors of teacher training programs in Spain. The results show statistically significant differences in the factors indicated, according to sex, age group, teaching experience, and experience with students who require educational support. According to the study results, a series of recommendations are included to improve the training necessary for university professors to promote inclusive education.

## 1. Introduction

The international community has urged governments through different world conferences to promote legislative and political frameworks under the principles of inclusive education. We have found examples of these initiatives since the 1990s [1,2]. For example, the 4th goal of the 2030 Agenda for Sustainable Development of the United Nations [3] establishes the need for a framework that guarantees inclusive and equitable quality education and promotes lifelong learning opportunities for all. This Sustainable Development Goal introduces measures to implement global commitments at both national and regional levels in this field. Despite all these recommendations and the actions developed, we cannot affirm, at present, that higher education is an inclusive space in which students with disabilities can develop under identical conditions.

Under the concept of disability, we include any physical, motor, intellectual, mental, or sensory impairment (auditory or visual) of permanent or temporary nature, limiting people’s ability to carry out activities of daily living. In addition, these limitations can be increased by economic and social conditions.

A disability paradigm is a restrictive approach because it is focused on the internal difficulties that people have. An inclusive model has replaced this approach. The inclusive paradigm shifts the attention to the environmental conditions and how they interact with the people.

In practice, this change allows us to overcome the restrictions of intervention models focused on personal limitations and rehabilitation. Following the inclusive paradigm, the intervention approach deals with personal limitations. Still, it considers their combination of social and environmental circumstances, physical, cognitive, social, and sensory barriers that determine daily activities. In this sense, the students’ disabilities or limitations are the conditions and resources that an environment offers them. Thus, students with a hearing disability may not have learning difficulties if they have appropriate devices to facilitate access to information.

We find the “special educational needs” (SEN) concept associated with the inclusive model in the educational field. This concept refers to students who have significantly different school performances from their classmates. Therefore, they need different or more educational resources to achieve their learning goals.

Inclusive education defends the right of people to learn in a standardized system that considers their needs, disabilities, and circumstances [4]. It is a continuous improvement process that identifies and eliminates barriers or factors that limit the possibilities of participation and educational success of all students [5,6].

Other concepts in close relation with the inclusive education paradigm are “universal accessibility” (UA) and the “universal design of learning” (UDL). UA refers to the flexible and open way of designing and constructing devices and buildings to be used by the majority of people, taking into account their particular circumstances [7]. The UDL aims to develop a flexible curriculum to adapt to the different educational needs of each learner. Therefore, educational institutions must provide teaching by seeking multiple means of representing and expressing knowledge and facilitating, as much as possible, the participation of “all” students in curricular and extracurricular activities, including those students with disabilities [8,9].

The inclusive concepts and principles have been gradually incorporated into compulsory education in the last decades. However, their incorporation into higher education is more recent and has not been applied in all its complexity. European universities have restructured their curriculum under the Bologna Process. The curricula have been restructured, according to the competencies of professional profiles; the variety of learning methods and assessment systems is expanded [10]. In addition, many European universities have developed designs with curricular flexibility to support people with disabilities.

### 1.1. Background

Despite the opportunity and the regulations adopted, the latest report on European higher education [8] indicates that few universities have implemented adequate measures to ensure physical and curricular accessibility for students with disabilities.

Dalton et al. [7] carried out a study in some universities in South Africa and the United States similar to the European study and recognized that inclusion and equity of access is, theoretically, a priority for universities. However, in practice, there is a need to continue supporting these institutions to achieve greater accessibility.

In Spain, the Olivenza Report [11] shows that 34.4% of the Spanish population has a higher education; this percentage is only 16.8% for people with disabilities. University students with disabilities currently make up only 1.7% of the total number of students. Several studies [12,13] confirm university students with disabilities’ difficulties in overcoming architectural barriers and access to information. Other studies [14,15] point out that the problems encountered have an impact on the self-concept and the perception of well-being and mental health of students with disabilities.

The Universia Foundation [16] has carried out several studies using data provided by university administrations. The results show the efforts and progress made by institutions to promote inclusion. In this sense, they highlight the creation of care services for students with disabilities and other specific needs, the adaptation of physical and architectural environments for accessing facilities, the provision of accessible transport, and specific human and material resources, according to their economic availability.

The *Universia Foundation* [16] reports indicate that there would be an average of 1.5% in the Spanish university student population with disabilities. Most college students with disabilities have a degree of disability between 33% and 64%. However, in 25% of cases, the universities do not report more than “exceeding 33%” (Spanish law considers disability to exist when a person reaches a degree equal to or greater than 33%). Regarding the disabilities, physical–organic disabilities are the most frequent, with an average of 51.7%; mental or psychological disorders represent 16.1%; visual disabilities 8.3%; hearing disabilities 7.2%. The proportion of university students with intellectual disabilities is low (1.2%). The remaining percentage (15.5%) is included under “other disabilities or combined disabilities.” By field of knowledge, many students with disabilities elect studies in Social and Legal Sciences (51%), while in science studies, they have the lowest presence (5.3%).

Other studies [17,18] have been collected from the students with disabilities to triangulate the information. Although the results are not conclusive and differ according to the type of disability and the methodology used in the study, the emphasis is on the demand for greater resources, greater accessibility to information, and precise curricular adjustments according to their needs.

Finally, professors [4,6,19] have a fundamental role in promoting inclusion and equity in learning. Professors’ attitudes towards disability and their influence on student performance are studied factors [20,21]. These studies suggest that attitudes towards integration are strongly influenced by the nature of the disabilities and educational problems being presented and, to a lesser extent, by the professional background of the respondents. Professors are optimistic about integrating only those students whose disabling characteristics are not likely to require extra instructional or management skills on the part of the teacher [21]. 

Professors’ attitudes towards integration reflect a lack of confidence in their instructional skills due to initial training received by future professors [22,23]. In line with this, a descriptive study [24] collects information about some adaptations that university professors carry out and the perception of their ability to carry out these adaptations. In this sense, the present study aims to understand the professors’ willingness to meet the needs of their students. The review of the scientific literature on the subject provides significant factors that facilitate the inclusion of university students with disabilities. These results indicate that social relationships, the access conditions to facilities and services, the attitude of classmates and professors, and personalized curricular adjustments are crucial elements in eliminating barriers to learning. However, based on the variety of factors found and the wide diversity of students’ needs, there is a need for more research within the university context to generalize results and be effective in the orientations.

### 1.2. Objective

Therefore, starting from the above points, the present work looks toward answering the following questions:

- Are there the necessary means and training available to carry out actions to enable the inclusion of SEN students in higher education institutions?
- Which are the main obstacles that university faculty find in implementing curricular adaptations?
- What kinds of adaptations are being made at the university to meet the needs of the SEN students?
- What is the opinion of university faculty on the inclusion (academic, social, etc.) of SEN students in the university?

To do that, we designed research of which the general aim is to analyze the difficulties that professors find when promoting inclusive education to offer training according to their needs. This objective is specified in the following specific objectives based on four dimensions of the teaching–learning process:

1. Detect the problems that the professor perceives in the accessibility to facilities and teaching resources.
2. Know the professors’ willingness before the specific needs of their students.
3. Describe the curricular adjustments professors say they perform to meet those needs.
4. Know the perceptions that professors have about the interactions between students with specific needs and their classmates and professors.
5. Analyze whether differences in the four dimensions studied are based on sex, age group, teaching experience, and experience with students who require educational support.
6. Propose, according to the results obtained, recommendations to address the permanent training of university professors.

## 2. Materials and Methods

### 2.1. Study Desing 

It is a non-experimental, descriptive, and association design between variables using non-parametric techniques.

### 2.2. Participants

The population under study has been the University of León (ULE) Education Faculties teaching staff in the Autonomous Community of Castilla y León and the Complutense University of Madrid (UCM) in the Community of Madrid. 

To guarantee the representativeness of the sample, both the total population (number of full-time professors who teach at the Faculty of Education of ULE or UCM) and the distribution among the different departments where they train within the Faculty of Education degrees (degree in Primary Education, degree in Early Childhood Education, and degree in Social Education) in addition to the Master’s Degree in Teacher Training for Compulsory Secondary Education and Baccalaureate, Vocational Training and Teaching of Languages, and the Master in Educational Guidance. Thus, out of a total of 179 professors at ULE and 175 professors at UCM, the sample was made up of 210 professors (112 from ULE and 98 from UCM), of which 117 are women and 93 men, whose distribution by departments is the one shown in Table 1.

The average number of students with disabilities in the UCM in the last ten years stands at 0.85%. In the ULE, the average percentage is 0.79% in the same time range. By field of knowledge, both in the UCM and in the ULE, the highest rate of registered students with disabilities is enrolled in the field of Social and Legal Sciences, 43% in the UCM, and 37% in the ULE. In the UCM, during the academic year 2018–2019, the students registered in the Office for the Inclusion of People with Diversity (OIPD) with a recognized disability certificate are distributed as 37% with a sensory disability, 28% due to physical-organic disability, 17% with mild developmental disorders (ADHD/ASD), 14% mental disability, and 4% mild intellectual disability. In addition, a total of 518 cases with other specific educational support needs are registered; among them, 25% of learning difficulties (dyslexia) stand out. The data provided by the ULE identify 31% physical–organic disability, 17% sensory, 6% psychic, and in the rest of the percentage, the cause is not specified.

In summary, the data provided by the universities under study show the same trend in terms of the kind and degree of disability of university students registered. 

The guidelines facilitated by the disability services of the universities, based on the type of students’ educational needs, should guide the adaptations that the professors make in their courses. In this case, the most common conditions are related to mobility, communication, and relationship. It is essential to highlight that due to the approach adopted in this study, in coherence with the explicit theoretical framework, we understand that the educational needs of the students are individual and the result of the interaction of multiple factors, not only of the etiology of their disability.

### 2.3. Instrument and Procedure 

The scale developed by Rodriguez-Martin and Alvarez-Arregui [25] was adopted, consisting of a Likert-type scale with four response options depending on the degree of agreement (from lowest to highest) with a series of items related to the educational response towards students with disabilities. The scale demonstrated adequate psychometric properties of reliability and validity. The internal consistency reliability analysis yielded a Cronbach’s α of 0.929. The validity analysis was performed using the AMOS extension of the SPSS statistical package, which revealed the excellent fit of the model to the four dimensions that form it (χ^2^ = 897.029, *p* < 0.000; CFI = 0.96; RMSEA = 0.63), according to Byrne’s criteria [26].

Our study used it as the first part with socio-academic identification items grouped by gender, age group, teaching experience, and previous experience with students in need of support and knowledge. In a second part, thirty-four items were grouped into four categories or factors: the first factor (FI) refers to accessibility and the universities’ resources to meet the student needs, formed by eight items. The second factor (FII) refers to the lecturers’ willingness regarding the students with special educational needs (SEN), created by ten items. The third factor (FIII) indicates the actual implementation of the professors’ curricular adjustments to meet the students with special educational needs (SEN), formed by eight items. Finally, the fourth category seeks to know the relationships of the SEN with their classmates and professors and their participation in the different subjects, formed by eight items. An open response space was incorporated at the end of each factor for teachers to make pertinent observations to exemplify or clarify their answers.

It was decided to maintain the response format with four Likert-type alternatives to “force” professors to choose between sides in the agreement. Due to social desirability when in doubt, the tendency is to select the “neutral” response.

The initial version had 39 items, but after carrying out the Spearman–Brown correction formula, it was decided to use only those items that correlated above 0.35 and clearly belonged to one of the four factors found. This is why the final questionnaire is made up of 34 items.

The questionnaire was managed from January to July 2019. In addition, an email was sent to the identified professors to explain them the study goals, request their collaboration to fill in the online questionnaire with Google form, and ask for their free consent to participate.

After filling in the questionnaire for the sample, the data analysis was carried out using the software package (SPSS version 25) and the free software JASP, according to the analysis technique used, as they were considered suitable resources for this research. As can be seen in Table 2, both alpha and omega indicate good internal reliability, since the values must range between 0.7 and 0.9 for both indexes to be considered adequate [27].

Although Cronbach’s alpha is the most commonly used statistic in the social sciences, we have used the McDonald’s omega following the recommendations of the American Psychological Association (APA) for ordinal Likert-polytomous scales.

## 3. Results

### 3.1. Sample

The sample was made up of 55.7% women and 44.3% men (Table 3). We emphasize that the highest percentage in age range corresponds, in 41% of the cases, to professors over 50, more than half of the respondent sample (61%) is over 46, denoting how the youngest teaching staff (less than 30) is the one with the slightest presence, specifically 6.7%. Regarding the seniority as university professors, it should be noted that more than half of those surveyed, 58.1%, have been working at the institution for more than 16 years, so we find a group with significant working experience. Only 20% have experience of fewer than five years. A significant percentage of the teaching staff, 58.6%, only identifies themselves as having worked with a range between 1 and 5 students with special educational needs. Finally, it should be noted that 49% of the teaching staff have not participated in any training activity related to inclusive education in the last five years.

### 3.2. Descriptive Analysis by Dimensions

In the first factor, the percentage of responses in this category indicates the need to improve practically all the items (Table 4). If we add the difficulties of an inaccessible environment to the individual needs of students with disabilities, it seems evident that the conditions and learning outcomes will be affected by these barriers.

Factor II allows for differentiating the intentionality of the teaching staff from their actual actions (Table 5). Here, we intend to analyze their sensitivity towards student diversity and their predisposition to respond to their needs. Items are collected, asking them for their thoughts about the curricular elements that should be modified, regardless of whether they used them or not. We emphasize that professors think that the objectives, contents, or evaluation criteria should not be adapted at this educational level. However, the activities, teaching resources, and teaching methods should be adapted. It is believed that there is not adequate coordination between the professors and the universities’ disability support units and, also, training would be needed to be able to face all the students’ different needs.

The third factor is about the adjustments in the different curricular elements that university professors do to respond to the specific needs of students. As shown in Table 6, the curricular elements with a higher percentage of the agreement allow more time for work deadlines or exams (item FIII.26), representing 83.3% approval (adding the categories A and TA). On the other hand, a slightly lower percentage of professors (81%) modify teaching resources, item FIII.22. Finally, on the opposite side, we highlight that a very high percentage of professors do not modify the contents of their subjects (70% adding categories TD and D, item FIII.20) nor adapt the objectives (69.1%, item FIII.19), summing the same categories.

Finally, Factor IV, the vast majority of professors consider that the relationships between students with disabilities and their classmates and professors are good (Table 7). There are volunteer colleagues in the classes to perform support tasks such as sharing notes and materials or integrating them into the working groups. On the other hand, it is recognized that students with disabilities have more difficulties carrying out practical activities and more significant academic difficulties. Regarding the perception of whether the awareness campaigns undertaken by the university are adequate or not, opinions are divided; 51.4% consider them adequate and 48.6% not adequate.

### 3.3. Analysis by Comparison of Groups in the Independent Variables 

In order to apply the appropriate analyzes, first, the Kolmogorov–Smirnov test with Lilliefors correction was performed to determine whether the data were normally distributed (null hypothesis of the analysis). When finding a significance of less than 0.05 in all the variables studied, the null hypothesis of normality was rejected, and non-parametric analyses were chosen. The Mann–Whitney U for the study of two independent groups (gender) and the Kruskal–Wallis H for the study of three or more independent groups (age teaching seniority, departments to which the professor belongs, and number of SEN students to whom he has taught) were used.

As the data do not follow a normal distribution, we do not work with means, but rather ranges are used to contrast the hypothesis that *K* samples have been obtained from the same population. It is a matter of contrasting whether the different independent samples are equally distributed and that, therefore, they belong to the same population, and it can be considered that compares medians.

#### 3.3.1. Comparison by Gender

Mann–Whitney U analysis was performed to check whether there are differences between men and women. Therefore, we opt for Mann–Whitney U analysis, since it allows us to compare the results of two independent groups (men and women in this case).

Table 8 reflect the items that present statistically significant differences grouped by factors. For example, men have a better perception of the classrooms space in order to adapt it to group work and, thus, the support of students with special education needs by their classmates (*p* = 0.005), the equipment of the classrooms (*p* = 0.003), and the existence of support technology (*p* = 0.037).

Women make more adaptations of the didactic material (*p* = 0.046), offer more time for exams (*p* = 0.003), and more frequently incorporate content related to the principles of universal learning design (*p* = 0.035). The magnitude of effect size is performed by the Hodges–Lehmann estimate, which uses the median differences between the two groups through the biserial rank correlation (r_b_), interpreting an irrelevant effect size if it is <0.1, small (0.1), medium (0.3), and large 0.5 [27]. In this case, we would find in all the items with statistically significant differences with an association strength between small and medium: (r_b_ = 0.21) in the perception of the classroom space; (r_b_ = 0.22) the adapted equipment; (r_b_ = 0.15) existence of support technology; adaptations of the didactic material (r_b_ = 0.11); more time to carry out exams and work deadlines (r_b_ = 0.22); in the incorporation of the UDL principles (r_b_ = 0.22).

#### 3.3.2. Comparison by Age Rank

In order to check whether there are statistically significant differences depending on the age ranges of the participants, a Kruskal–Wallis analysis was carried out with the seven independent age groups. The results only show differences in two variables: (FI.5) the equipment that classrooms have is adapted to attend SEN student (*p* = 0.015); in item FII.18, the need for training in universal design of learning (*p* = 0.010). Those who indicate a greater need for training are professors over 50; professors between 31 and 35 are those who show the slightest need. As they are categorical variables, the strength of the effect size is performed using Cramer’s V, and its interpretation is: irrelevant effect size if <0.1, small (0.1), moderate (0.3), and large 0.5 [28]. The effect size was calculated for the pairs significantly different in the post hoc analysis between each pair of groups, with significance correction through the Mann–Whitney test. In item FI.5, we highlight that the age group that observes a minor classroom adaptation is those over 50 with an effect size of 0.25. In item FII.18, we also find an effect size of 0.23 for the group over 50.

#### 3.3.3. Comparison by Teaching Seniority

We find more statistically significant differences if we take into account the seniority of professors (Table 9). Through a Kruskal–Wallis analysis with five independent groups (1 = less than 5 years; 2 = between 6 and 15 years; 3 = between 16 and 20 years; 4 = between 21 and 30 years; 5 = more than 31 years of teaching), we obtained the results shown in the table below.

It can be seen how professors between 21 and 30 years of teaching experience perceive fewer architectural barriers, less need for training in the universal design of learning, and think awareness campaigns are adequate. On the other hand, professors in the range between 6 and 15 years perceive awareness campaigns to be less adequate. Those between 16 and 20 years perceive architectural barriers, and those who are less than 5 years perceive a greater need for training at the UDL.

On the other hand, professors between 6 and 15 years of teaching experience perceive greater participation of SEN students in classes and a better relationship between them and the other students. In addition, they are the ones who adapt more material in order to meet the students’ needs. However, professors with less than 5 years of teaching experience have the worst scores in these three aspects.

Finally, professors with more than 30 years of teaching seniority perceive more support technology in the classrooms to help SEN students, while professors between 6 and 15 observe more deficiencies in this area. Professors with fewer years perceive fewer SEN students’ classmates who carry out support and volunteer activities. The strength of the effect size in all the items would be found, using Cramer’s V, between a small effect (V = 0.1) and almost moderate (V = 0.27).

Post hoc analyses (Table 10 and Table 11) show differences in almost all the factors evaluated between groups 1 and 2 (professors’ seniority: less than five years and between 6 and 15 years) and groups 2 and 4 (professors’ seniority: between 6 and 15 years and between 21 and 30 years), concerning the items where the Kruskal–Wallis test is significant. However, no significant differences are found between groups 3 and 5 (professors’ seniority: between 16 and 20 years and more than 31 years) and groups 4 and 5 (professors’ seniority: between 21 and 30 years and more than 31 years). Therefore, it indicates that after 20 years of teaching experience, perceptions about the inclusion of students become homogenized.

To know the strength of the effect size in all the items, we use Cramer’s V. In all the cases, it observes a small (V = 0.1) or moderate (V = 0.3) effect. We highlight FI.1. (V = 0.27), the 6–15 years group vs. the 21–30 years group and also with medium effect size (V = 0.26) the item FI.6. in the same group; FI.18. (V = 0.16) in the pair 5 vs. 21–30 years; in FIII.22., the groups 5 vs. 21–30 years (V = 0.15); FIV.27. (V = 0.1 8); IVF.30. (V = 0.15); IVF.31 (V = 0.17, in the first two groups; IVF.34 (V = 0.14) in the third group.

#### 3.3.4. Comparison of Results by Area of Knowledge

From the analysis, according to the area of knowledge shown in Table 12, we highlight the following differences by factors.

In factor I, referring to the percentage of more remarkable agreement on the buildings’ accessibility and the resources provided by universities, we highlight that Law professors perceive these conditions to be more favorable. On the other hand, those who indicate a greater need for auxiliary support personnel are from Biomedical Sciences.

Factor II on the disposition of professors, the teaching staff of Pedagogy show greater training in the items related to the universal design of learning, while Law professors demand more specific training. Experimental Sciences professors say that they know the UDL less but do not see the need for training. Biomedical Sciences are the ones that have the slightest need to modify their objectives and methodology in subjects.

In factor III, referring to the actual implementation of the adjustments in the subject curricular elements, Psychology professors are the ones who say they make more methodological modifications aimed at SEN students, complementing the learning with tutorials. On the other hand, together with their disposition and thoughts, those in Biomedical Sciences are the ones that less often adjust the objectives and methodologies in their subjects. However, Philology and Art professors claim to incorporate more content of this type in their teaching.

Finally, factor IV refers to the relationships between students and professors. Experimental Sciences professors, in a higher percentage, perceive that there are good relations between all of them. Those who perceive a greater disagreement with the statement are Law professors. Those who have the least knowledge about the figure of the volunteer student supporting SEN students are the Experimental Sciences professors. The effect size is slightly larger in almost all the items than the one found in the preceding factors, close to moderate strength of association between items and the highlighted areas of knowledge. In factor I: item 5 (V = 0.20); item 6 (V = 0.25); item 7 (V = 0.24). Factor II: item 14 (V = 0.23); item 16 (V = 0.34); item 17 (V = 0.25). Factor IV: item 28 (V = 0.23) and item 34 (V = 0.24). In items 10, 18, 20, and 23, the strength of association is considered irrelevant.

The post hoc analyses show no substantial differences between the two main areas of knowledge of the Faculty of Education: Psychology and Pedagogy. However, they do offer significant differences between these areas with Law’s (Table 13 and Table 14).

These results show that Law professors are less aware of the problems related to accessibility and access to resources than their colleagues in Psychology and Pedagogy. Along the same lines, Law professors think that the goals or even the contents of the courses should be adapted, but without the need to modify the methodology or to include UDL contents or activities. Likewise, the perception of the Psychology and Pedagogy professors about the relationship between students and the professor is better than that of the professionals in the area of Law. The effect size is slightly larger in almost all the items than the one found in the previous factors, with a nearly moderate strength of association between items and the highlighted areas of knowledge. In factor I: item 5 (V = 0.20); item 6 (V = 0.25 pair Psy-Law); item 7 (V = 0.24). Factor II: item 14 (V = 0.23); item 16 (V = 0.34); item 17 (V = 0.25). Factor IV: item 28 (V = 0.23) and item 34 (V = 0.24). In items 10, 18, 20, and 23, the association’s strength is considered not relevant.

#### 3.3.5. Comparison of Results According to Number of SEN Students Attended by Professors

When we compare the perception of professors based on SEN students who have taught throughout their teaching career, through a Kruskal–Wallis analysis with five independent groups (1 = none, 2 = between 1 and 5 students, 3 = between 6 and 10 students, 4 = between 10 and 15 students, and 5 = more than 15 students) the results shown in Table 15 are obtained.

Professors who have not had the opportunity to teach any SEN student in their classes seem to be the ones who have the worst perception about the classroom equipment for the inclusion of SEN student (within Factor 1 of accessibility and resources). In addition, there are those who claim to have less knowledge about the UDL and worse perception of the relationship between students with and without SEN (within Factor 2 of professors willingness to meet the needs). It also seems contradictory that they claim to adapt the practical activities more than those professors who have had between 6 and 15 SEN students, since they have not had any.

The professors with the highest number of SEN students seem to make the most modifications in their practical classes and have the best perception about the classroom’s equipment for the inclusion of SEN students.

Finally, the professors who have had between 6 and 10 students and between 11 and 15 SEN students are those who have better perceptions in the Factor 2 items, where significant differences have been found between groups: the relationship between students with and without SEN and the knowledge about the universal design of learning, respectively.

From the results of the post hoc analyses of the Kruskal–Wallis test, it can be inferred that there are significant differences between all groups concerning group 5 (professors who have had more than 15 SEN students). These differences are about their perception of the adaptations of classroom equipment (FI.6), with teachers in group 5 being those who perceive more adaptations of equipment. The strength of the effect size for group 5 is medium (V = 0.3).

Similarly, teachers who have had between 11 and 15 SEN students show a broader knowledge of UDL (FII.16) than those who have had none or have had between 1 and 5 or between 6 and 10 SEN students. The strength of the effect size for the group that had between 11 and 15 SEN students is V = 0.2.

In the same way, significant differences concerning the perception of students’ relationships with special educational needs with the rest of their classmates (FVI.27) were found. Again, these differences are present between groups 1 and 3 (no students vs. 6–10 students) (*p* = 0.010) in favor of teachers who have had between 6 and 10 SEN students; between groups 2 and 3 (between 1 and 5 vs. 6–10 students) (*p* = 0.020) in favor, again, of teachers who have had between 6 and 10 SEN students; between groups 3 and 4 (between 6 and 10 students vs. 11–15 students) (*p* = 0.029) in favor of those who have had more SEN students (group 4). In this case, we found effect size strengths between the groups ranging from V = 0.17 to V = 0.29, i.e., small to moderate strengths.

## 4. Discussion

Nowadays, there is a growing concern about students with disabilities, since thousands of young people with disabilities lag in educational development indicators; hence, there is a need to implement studies that confirm the effectiveness of the actions developed with students with disabilities. In this sense, the general aim of the present research is to analyze the difficulties that professors find in promoting inclusive education to offer training according to students’ needs. This objective is achieved, as information is obtained about the specific objectives that are presented below. 

First, related to problems perceives by professors about the accessibility to the facilities and teaching resources, the results show that there is agreement among the teaching staff regarding the need to improve accessibility and the resources offered by the university to facilitate inclusion: from mobility and access to the classrooms, the adaptation of classroom equipment, support of technologies for students with special educational needs to counting on auxiliary support staff. The contributions of the teaching staff in the first factor in the open-ended responses report specific issues of infrastructure and personal resources that would need to be improved. For example, they stated that: “the spaces do not favor mobility and make access difficult for people who need to use wheelchairs, the access corridors to classrooms and professors’ offices are narrow”; “most of the classrooms have fixed tables and chairs anchored to the floor, which are a barrier for students with mobility problems”; “the platforms and classroom furniture hinder the passage of students with motor and visual difficulties”; “there is no signage to identify spaces in Braille or relief code”; “more support staff or a specific service would be needed both to adapt materials (videos, presentations or class notes) and to support the use and maintenance of specific materials for people with visual and hearing impairment”; “there are FM microphones for selective sound amplification, but their maintenance is not always adequate.”

This shows that facing disability from an inclusive paradigm at the universities requires a workforce trained and equipped with skills to work in a cross-disciplinary manner to operationalize the actuation guidelines designed to promote the delivery of educative services for students with disabilities [29]. 

Second, concerning professors’ willingness to respond to the specific needs of their students, it is necessary to highlight that the professors who have not taught students with disabilities have the worst perception of the resources and equipment of the classroom to provide inclusive education. In contrast, those who have worked for students with disabilities are the ones who most positively value the equipment in the classrooms and who have made the most significant number of adaptations to their teaching and learning processes. This study confirms that professors are an essential factor for the performance improvement of university students with disabilities [30,31]. The initial and in-service teacher training programs should include issues related to the diversification of teaching and assessment methods and incorporating inclusive pedagogy. With this, it pushes the creation of environments rich in learning opportunities for all students, recognition of individual differences, and developing competencies for collaborative work. 

Active approaches to learning (cooperative learning, situated learning, service learning, or project-based learning) should be incorporated into teacher training, fostering increasingly inclusive environments [32]. The adoption by professors of an appropriate methodology is a better guarantee that the inclusive process will become a reality.

Additionally, as mentioned in previous research [33], education resources based on information and communication technologies (ICT) are considered valuable instruments by the professors. In this sense, the data collected in this regard in the open-ended responses report that “professors can deliver online materials (course notes, exercises, etc.) before the sessions if they have the support of specialized resources and personnel.” This allows students with or without disabilities to prepare for the course sessions. Furthermore, they remark “that universities should be equipped with scanners given the extensive use of scanning and OCR by students with all types of disabilities”; “The use of scanners combined with laptops or tablets for the students increases the accessibility to the learning materials.” Besides, the use of ICT facilitates another UDL: “allowing the online submission of the assignments, some students have great difficulties getting to the professor’s office due to their medical situation or the lack of accessibility of the campus”; “QR with accessible texts to expand information on schedules and conditions of use of services and facilities.”

Third, related to curricular adaptations that professors declare that they make to meet students’ needs, the present study shows that the measure most used for students with disabilities allows for more time for work deadlines or exams, followed by modifying teaching resources [13]. Thus, the changes professors make to adapt the training process to students with disabilities do not imply any meaningful or substantive modification in the teaching–learning process [34]. In this sense, among the professors surveyed in the area of Law, there is evidence of the perception that it is necessary to adapt the objectives and contents, but not the methodology. This contrasts with the view of professors in other areas such as Psychology and Pedagogy, which may be due to the specific training in the inclusion of this group. In this regard, it is essential to emphasize that inclusion implies the restructuring of the teaching practices themselves [35]. Higher education institutions and quality assurance agencies should recognize and encourage the development of inclusive practices to become more widespread. In this regard, teacher promotion programs and degree accreditation could include indicators that acknowledge the development of measures and resources to favor all students’ physical and curricular accessibility to learning.

Future research should triangulate these results with those provided by students with disabilities to understand their perspective. The future assessment and planning processes must include the students’ experiences with disabilities in future assessment and planning processes. It is only then that educative and social change can enhance their participation [20,34].

Fourth, regarding the lecturers’ perceptions about the interactions between SEN students and their classmates and professors, previous studies [34] confirm that this is one of the factors that most influences the development of students with disabilities. The present study shows a positive perception by university professors about the adequate participation and relationship of students with disabilities in the classroom, with their classmates, and with the teaching staff. However, previous studies coincide in good relationships with classmates and professors, but students state that professors do not have enough training to respond to their needs [13,22]. Social integration and good relationships with peers allow students to solve many academic difficulties derived from their disability. For example, they solve doubts about subject contents and obtain materials and notes through their peers, highlighting peer learning and mentoring as essential resources. Thus, in the case of students with disabilities, they can improve the learning and integration process in university education. As shown in the results, professors in Pedagogy and Psychology consider that the relationships between students with disabilities and professors are good, to a greater extent than the assessment made by other colleagues. This may be due to the positive evaluation developed by these professors in terms of the number of interactions, tutorials, and support situations in which they have responded to students with disabilities. Pedagogy and Psychology professors’ quantitative and qualitative responses report that their interactions with students with disabilities are more favorable than professors in other areas. This tendency could be interpreted in the sense that greater knowledge and closeness to the individual needs of these students leads to more satisfaction in the faculty and better performance of the students [36]. 

In fifth place, we studied the differences between the four dimensions considered based on sex, age group, teaching experience, and experience with students who require educational support. The results show that male professors have a better perception of accessibility and university resources for students with disabilities than female professors. Nonetheless, female professors show a better willingness to meet the students with disability needs. These results are in line with the findings, which indicate that women are more prone to identify curricular or architectural obstacles that students with disabilities must overcome [37] and have greater proactivity towards providing accommodations and adopting UDL principles [34]. These results can be explained in terms of variables such as empathy, since women have always obtained higher scores in this sense than men. Additionally, it could be because women have been traditionally socialized in nurturing and caring roles, which may explain differences in male and female attitudes. However, any gender differences should be interpreted with caution due to the characteristics of the sample used [38].

Age does not seem to be a differentiating variable in terms of perception of the inclusion situation (accessibility and resources, teachers’ disposition, curricular adjustments, and SEN students’ relationships). Nevertheless, years of teaching experience does seem to be differentiating. The professors with more experience perceive greater accessibility and university resources for students with disabilities and show a greater need for training in the UDL. This circumstance has also been appreciated in previous studies [7]. It denotes that, despite the high levels of stress and burnout [39] among university professors, educational inclusion is not affected by years of professional experience. The commitment to inclusion is more present in the professors who are developing their professional careers (experience between 6 and 15 years). They are the ones who are making the most significant number of curricular adaptations. This commitment may be due to their consolidation in the profession and the fact that they have had previous training in educational inclusion and need to achieve tangible and not theoretical equity both at the social and educational level.

## 5. Conclusions

According to the results obtained, we have to make several recommendations to guide future interventions and address university professors’ permanent training [40]. The first conclusion is that there is a general lack of knowledge about the existence and functioning of the care and support services for people with disabilities in universities. It proposes awareness campaigns focused primarily on identified target groups to raise awareness of specific needs and make particular needs visible. These services have begun to develop in recent years, coinciding with the increase in the number of people with special needs at the university. However, their resources, actions, and functions are not always well defined or known by the faculty [41]. The faculty should make an effort to know and take part in the activities of these services. In addition, the staff of these services must have enough training and resources to support or suggest the curriculum adaptations that SEN students require. 

These services can become integration points, offering information about resources, training options, and links between education and employment to move forward in a university inclusive educational response to diversity [20,42]. In this sense, it is proposed to establish coordination mechanisms between disability support services and course or degree coordinators. Our results show how most professors (49%) have not carried out any training activities concerning SEN students’ support or the UDL in the last five years. A significant percentage of faculty are unaware of the principles and ways to UDL’s implementation. The professors require support to recognize the difficulties of access and participation of students with disabilities, especially male professors, who have less capacity than their female colleagues to identify these needs and barriers. Based on the results, we propose the development of program training courses on specific knowledge of the different types of disabilities; on curriculum training in design for all for teachers of varying university degrees to introduce the concepts of universal accessibility (UA) and universal design for learning (UDL); knowledge of UDL principles and their practical implications: how to provide multiple means of participation, representation, action, and expression of learning. Similarly, emphasis should be placed on training in active methodologies with multilevel activities, authentic assessment tools, and resources (e.g., how to assess by competencies and not by content or learning rubrics); information and concrete practices on how to make essential subject documentation (word texts, presentations, and videos) accessible to all students. If the training programs link with the tenure track or promotion of the professors, they would have more success.

In this regard, the teaching training program and its recognition for professors should be complemented at a macro level (institution or system). In this way, we propose the incorporation of quality indicators related to the degree of attention to disability and accessibility that the degrees of the universities or the universities themselves have.

It should change the orientation that most of these services have. Most of them are working under a care-based approach instead of adopting the inclusive paradigm, which is more feasible in the education field. They should offer services focused on rights and the promotion of the abilities of the SEN students [20]. These changes would allow the services to contribute to the professional development of the university faculty, offering formal and structured training programs as well as recognizing their actions for inclusion. 

We emphasize that the statistical significance and the strength of the association between variables do not correspond to the importance of the practical applications, especially in the educational sciences. Nevertheless, the small and medium effects found in the items of the four factors may accumulate, producing a critical beneficial impact on the learning conditions of university SEN students. 

Joining this change to the previous recognition program, we conclude that the integration of the training activities and the recognition of the curricular adaptions developed as part of the promotion scheme would entail a greater interest and a greater willingness to care for SEN students.

The study presents some limitations. Although the results of this study are positive, the data are partial and should be completed. Furthermore, this was a cross-sectional study in which data collection was carried out in a single temporal moment. In addition, self-report data from professors joined to other measures of inclusive education at university for students with disabilities should be included in future researches. It would also be helpful to conduct more studies on how to measure disability-inclusive development in higher education.

## Figures and Tables

**Table 1:** Sample.

	ULE		UCM		Total
	Male	Female	Male	Female	
Biomedical Sciences	2	5	0	0	7
Law	19	20	11	7	57
Experimental sciences	0	4	1	3	8
Philology	2	5	5	3	15
Pedagogy	8	15	30	28	81
Psychology	10	22	5	5	42
Total	41	71	52	46	210

**Table 2:** Scale reliability statistic.

	McDonald’s ω	Cronbach’s α	95.0% Confidence Interval	
			Lower	Upper
Scale	0.850	0.859	0.830	0.885
FI	0.670	0.650	0.630	0.680
FII	0.750	0.710	0.640	0.753
FIII	0.810	0.790	0.750	0.835
FIV	0.685	0.679	0.730	0.755

*Note.* Of the observations, 210 were used, 0 were excluded listwise, and 210 were provided.

**Table 3:** Sample description.

		F	%
Genre	Male	93	44.3
	Female	117	55.7
Age	Less than 25 years old	2	1
	Between 26 and 30 years old	12	5.7
	Between 31 and 35 years old	16	7.6
	Between 36 and 40 years old	18	8.6
	Between 41 and 45 years old	34	16.2
	Between 46 and 50 years old	42	20
	More than 50 years	86	41
Time as a lecturer at the university.	Less than 5 years	42	20
	Between 6 and 15 years	46	21.9
	Between 16 and 20 years	34	16.2
	Between 21 and 30 years	60	28.6
	More than 30 years	28	13.3
How many students with educational needs have you taught?	None	18	8.6
	Between 1 and 5 students	123	58.6
	Between 6 and 10 students	45	21.4
	Between 11 and 15 students	11	5.2
	More than 16 students	13	6.2
Number of training activities related to the support of students with educational needs in the last five years.	None	103	49
	Between 1 and 5 activities	79	37.6
	Between 6 and 10 activities	12	5.7
	More than 10 activities	16	7.6

**Table 4:** Factor I. Accessibility and resources that universities have in order to facilitate inclusion (percentage of responses).

	** TD	D	A	TA
FI.1. There are architectural barriers *.	10.5	31.9	32.4	25.2
FI.2. Public transport is provided.	6.2	22.4	53.8	17.6
FI.3. Classrooms favor access/mobility.	27.6	44.8	23.3	4.3
FI.4. Classroom space allows group work.	32.9	45.7	16.2	5.2
FI.5. Classroom equipment is adapted.	33.8	47.6	15.2	3.3
FI.6. There are SEN support technologies.	42.4	41.9	11.9	3.8
FI.7. Auxiliary support staff is needed.	4.8	16.7	49.5	29
FI.8. SEN does not have facilities to carry out external internships.	4.8	31	43.8	20.5

* Item analyzed inversely. ** TD: Totally Disagree; D: Disagree; A: Agree; and TA: Totally Agree.

**Table 5:** Factor II: Professors’ willingness to meet the needs (percentage of responses).

	** TD	D	A	TA
FII.9. There is coordination between university support services-professors.	15.7	43.3	36.2	4.8
FII.10. Professors must modify the objectives of their courses.	35.2	34.8	23.3	6.7
FII.11. Professors must modify/delete the CONTENTS of their courses.	32.9	42.4	19.5	5.2
FII.12. Professors must adapt the ACTIVITIES of their courses.	7.1	22.4	46.2	24.3
FII.13. Professors must adapt the didactic MATERIALS.	4.8	14.3	46.7	34.3
FII.14. Professors must adapt the METHODOLOGY.	4.8	14.8	48.6	31.9
FII.15. Professors must adapt the evaluation system.	9	23.8	43.8	23.3
FII.16. I know the UDL *.	37.6	27.6	20	8.23
FII.17. In my courses I include contents/activities related to the UDL.	14.3	32.4	29.5	23.8
FII.18. I need training to introduce UDL *.	4.3	9.5	41.9	43.8

* UDL = universal design of learning. ** TD: Totally Disagree; D: Disagree; A: Agree; and TA: Totally Agree.

**Table 6:** Factor III Real implementation of the curricular adjustments (percentage of responses).

	** TD	D	A	TA
FIII.19. In my classes I adapt the objectives of my subject to students with special educational needs.	32.4	36.7	24.8	6.2
FIII.20. In my classes I modify the contents to student with special educational needs.	31.4	38.6	22.9	7.1
FIII.21. In my classes I make modifications and/or deletions activities.	9.5	15.2	52.4	22.9
FIII.22. In my classes I modify the didactic resources for students with special educational needs.	6.7	14.8	47.6	31
FIII.23. In my classes I modify the methodology to students with special educational needs.	7.1	18.1	46.7	28.1
FIII.24. In my classes I modify the Instruments of evaluation for students with special educational needs.	11	15.7	47.6	25.7
FIII.25. I adapt the practical activities to students with special educational needs.	5.2	13.8	44.3	36.7
FIII.26. Students with special educational needs have more time for exams/work deadlines.	5.7	11	33.3	50

** TD: Totally Disagree; D: Disagree; A: Agree; and TA: Totally Agree.

**Table 7:** Factor IV: Relationships and participation of students with disabilities and other classmates-lecturers (percentage of responses).

	** TD	D	A	TA
FIV.27. The relationship between students with special educational needs and their classmates is good.	1.4	9.5	48.1	41
FIV.28. My relationship as a lecturer with students with special educational needs is good.	0	1.4	32.9	65.7
FIV.29. In general professor–students with special educational needs communication is fluid.	0	12.4	53.3	34.3
FIV.30. Diversity awareness campaigns are adequate.	10.5	38.1	43.3	8.1
FIV.31. Students with special educational needs participate in all my subject activities.	1	9.5	36.2	53.3
FIV.32. Students with special educational needs have more difficulties in practices (laboratories. outside the university).	27.6	42.9	19.5	10
FIV.33. In my class, students with special educational needs have the same academic problems as their classmates.	5.7	28.6	39	26.7
FIV.34. There are volunteer students for support tasks.	4.8	31	43.8	20.5

** TD: Totally Disagree; D: Disagree; A: Agree; and TA: Totally Agree.

**Table 8:** Mann–Whitney U analysis to compare results according to gender.

Variable	Male		Female		Z	*p*
	N	Rank	N	Rank		
*FI. University accessibility and resources.*						
FI.4. Classroom space allows group work.	93	117.80	117	95.73	−2.811	0.005
FI.5. Classroom equipment is adapted.	93	118.52	117	95.15	−3.002	0.003
FI.6. There are SEN students support technologies.	93	114.53	117	98.32	−2.083	0.037
*FII. Professors’ willingness to meet students’ needs.*						
FII.17. In my subjects I include UDL content/activities.	93	96.37	117	112.76	−2.113	0.035
*Factor III. Current implementation of curricular adjustments.*						
FIII.22. In my classes I modify the didactic resources.	93	96.81	117	112.41	−1.998	0.046
FIII.26. SEN students have more time for exams/work deadlines.	93	92.80	117	115.54	−2.937	0.003
*Factor IV. Relationships and participation of students with disabilities.*						
FIV.27. Relationships between SEN students and their classmates are good.	93	118.35	117	95.28	−3.020	0.003

**Table 9:** Kruskal–Wallis analysis by sections of teaching seniority.

	Teaching Seniority Range (Years)					H	*p*
	<5	6–15	16–20	21–30	+31		
*Factor I. University accessibility and resources.*							
FI.1. There are architectural barriers.	114.17	89.46	83.84	123.42	106.77	14.85	0.005
FI.6. There are SEN support technologies.	107.86	83.87	107.31	112.49	120.32	9.88	0.043
*Factor II. Willingness towards disability.*							
FII.18. I need training in the UDL.	81.21	98.47	113.53	119.77	109.57	13.64	0.009
*Factor III. Current implementation of curricular adjustments.*							
FIII.22. In my classes I modify the didactic resources.	93.07	125.07	112.59	99.82	95.57	9.66	0.047
*Factor IV. Relationships and participation of students with disabilities.*							
FIV.27. Relationships between SEN students and their classmates are good.	84.71	117.63	118.50	103.47	105.32	12.03	0.033
FIV.30. Diversity awareness campaigns are adequate.	89.30	126.25	94.21	104.34	111.91	10.11	0.017
FIV.31. SEN students take part in all my subject activities.	105.00	91.78	96.47	123.78	100.59	10.46	0.039
FIV.34. There are support volunteer students.	130.64	104.46	95.65	94.76	104.48	13.63	0.025

**Table 10:** Post hoc Kruskal–Wallis analysis by sections of teaching seniority.

	<5 vs. 6–15 Years **			<5 vs. 16–20 Years **			<5 vs. 21–30 Years **			<5 vs. +31 **		
	*p*	<5 r	6–15 r	*p*	<5 r	16–20 r	*p*	<5 r	21–30 r	*p*	<5 r	+31 r
*Factor I. University accessibility and resources.*												
FI.1	0.045	49.9	39.5	0.019	43.6	32.1						
FI.6	0.037	49.8	39.5									
*Factor II. Willingness towards disability.*												
FII.18				0.009	33.1	45.1	0.001	40.4	59.2	0.045	31.5	40.3
*Factor III. Current implementation of curricular adjustments.*												
FIII.22	0.005	37.2	51.1									
*Factor IV. Relationships and participation of students with disabilities.*												
FIV.27	0.002	36.3	51.9									
FIV.30												
FIV.31	0.005	37.3	51.0	0.007	32.9	45.3						
FIV.34	0.032	50.2	39.2	0.009	44.1	31.5	0.002	61.9	44.1			

** N < 5 = 42; N 6–15 years = 46; N 16–20 years = 34; N 21–30 years = 60; N +31 years = 28.

**Table 11:** Post hoc Kruskal–Wallis analysis by sections of teaching seniority.

	6–15 vs. 16–20 Years **			6–15 vs. 21–30 Years **			6–15 vs. +31 Years **			16–20 vs. 21–30 Years **		
	*p*	6 –15 r	16–20 r	*p*	6–15 r	21–30 r	*p*	6–15 r	+31 r	*p*	16–20 r	21–30 r
*Factor I. University accessibility and resources.*												
FI.1				0.005	44.3	60.5				0.002	36.3	53.8
FI.6				0.009	45.3	59.7	0.008	32.7	45.2			
*Factor II. Willingness towards disability.*												
FII.18				0.046	47.2	58.2						
*Factor III. Current implementation of curricular adjustments.*												
FIII.22				0.038	60.0	48.4	0.013	41.7	30.4			
*Factor IV. Relationships and participation of students with disabilities.*												
FIV.27	0.014	45.4	33.7	0.041	59.7	48.6						
FIV.30				0.005	44.6	60.3				0.023	39.6	51.9
FIV.31												
FIV.34												

** N < 5 = 42; N 6–15 years = 46; N 16–20 years = 34; N 21–30 years = 60; N +31 years = 28.

**Table 12:** Kruskal–Wallis analysis according to the teaching area.

Variable	Teaching Expertise Area *						H	*p*
	Ped ***	Psy ***	Law ***	Phi ***	Bio ***	Exp ***		
*Factor I. University accessibility and resources.*								
FI.4. Classroom space allows group work.	95.03	95.89	125.70	115.83	121.38	84.38	13.48	0.019
FI.5. Adapted classroom equipment.	95.06	109.00	126.05	79.00	137.57	68.06	19.88	0.001
FI.6. There are SEN support technologies.	98.35	81.40	130.24	111.53	103.64	118.44	20.84	0.001
FI.7. Auxiliary staff is needed.	113.26	118.07	77.61	114.87	144.64	107.81	21.66	0.001
*Factor II. Professors’ willingness to meet students’ needs.*								
FII.10. Lecturer should modify the objectives of their subject.	96.48	110.19	121.98	104.03	58.50	98.69	11.69	0.039
FII.14. Lecturer should adapt the methodology.	114.10	119.39	89.42	92.17	82.29	105.31	11.25	0.047
FII.16. I know the UDL.	133.60	92.36	95.07	87.77	56.93	40.00	39.38	0.000
FII.17. I include UDL content/activities.	122.06	111.43	73.37	139.27	82.14	92.75	33.58	0.000
FII.18. I need training in the UDL.	109.35	94.85	118.69	82.50	91.50	69.00	11.6	0.041
*Factor III. Current implementation of curricular adjustments.*								
FIII.20. I modify the contents	93.44	109.75	122.58	115.30	75.50	91.44	11.59	0.041
FIII.23. I modify the methodology	105.14	128.90	91.00	117.83	71.07	96.63	14.38	0.013
*Factor IV. Relationships and participation of students with disabilities.*								
FIV.28. My SEN students-lecturer relationship is good.	107.00	124.25	85.13	113.90	97.14	128.56	17.66	0.003
FIV.34. There are support volunteer students.	106.75	116.71	104.39	116.73	75.14	47.44	12.3	0.031

* Ped: Pedagogy; Psy: Psychology; Law: Law; Phi: Philology, Art and History; Bio: Biomedical Sciences; and Exp: Experimental Sciences. *** N Ped = 81; N Psy = 42; N Law = 57; N Phi= 15; N Bio = 7; Exp = 8.

**Table 13:** Post hoc Kruskal–Wallis analysis analysis according to the teaching area (Pedagogy vs. others).

	Ped. vs. Psy.			Ped. vs. Law			Ped. vs. Bio.			Ped vs. Exp.		
	*p*	Ped r	Psy r	*p*	Ped r	Law r	*p*	Ped r	Bio r	*p*	Ped r	Exp r
*Factor I. University accessibility and resources.*												
FI.4.				0.002	61.1	81.4						
FI.5.				0.002	61.1	81.3						
FI.6.				0.001	60.6	82.0						
FI.7.				0.001	78.5	56.7						
*Factor II. Professors’ willingness to meet students’ needs.*												
FII.10.				0.01	62.5	79.4						
FII.14.				0.013	76.0	60.1						
FII.16.	0.00	70.5	45.5	0.000	80.5	53.8	0.004	46.7	19.0	0.000	47.9	15.0
FII.17.				0.000	82.7	50.7	0.002	46.8	17.0			
FII.18.												
*Factor III. Current implementation of curricular adjustments.*												
FIII.20.				0.004	61.6	80.6						
FIII.23.	0.02	57.0	71.5									
*Factor IV. Relationships and participation of students with disabilities.*												
FIV.28.				0.014	75.5	60.9						
FIV.34.												

Ped: Pedagogy (N=81); Psy: Psychology (N = 42); Law: Law (N = 57); Phi: Philology, Art and History (N = 15); Bio: Biomedical Sciences (N = 7); and Exp: Experimental Sciences (N = 8).

**Table 14:** Post hoc Kruskal–Wallis analysis analysis according to the teaching area (Psycology vs. others).

	Psy vs. Law			Psy vs. Phi			Psy vs. Bio			Psy vs. Exp		
	*p*	Psy r	Law r	*p*	Psy r	Phi r	*p*	Psy r	Bio r	*p*	Psy r	Exp r
*Factor I. University accessibility and resources.*												
FI.4.	0.01	41.9	55.96									
FI.5.										0.037	27.17	16.75
FI.6.	0.00	37.62	59.12	0.049	26.71	35.40						
FI.7.	0.00	61.65	41.41									
*Factor II. Professors’ willingness to meet students’ needs.*												
FII.10.							0.035	26.65	15.07			
FII.14.	0.00	58.32	43.87									
FII.16.										0.004	27.88	13.00
FII.17.	0.00	60.14	42.53									
FII.18.	0.03	43.35	54.90									
*Factor III. Current implementation of curricular adjustments.*												
FIII.20.												
FIII.23.	0.00		42.65				0.023	26.76	14.43			
*Factor IV. Relationships and participation of students with disabilities.*												
FIV.28.	0.00	60.5	42.26									
FIV.34.												

Ped: Pedagogy (N = 81); Psy: Psychology (N = 42); Law: Law (N = 57); Phi: Philology, Art and History (N = 15); Bio: Biomedical Sciences (N = 7); and Exp: Experimental Sciences (N = 8).

**Table 15:** Kruskal–Wallis analysis analysis according to number of SEN students supported by each professor.

	Number of SEN Students Supported by Each Professor					H	*p*
	None *	1–5 *	6–10 *	11–15 *	+15 *		
*Factor I. University accessibility and resources.*							
FI.5. Adapted classroom equipment	91.02	104.27	100.5	105.95	152.88	10.78	0.029
*Factor II. Professors’ willingness to meet students’ needs.*							
FII.16. I Know the UDL	78.03	103.59	107.90	158.14	108.77	13.29	0.01
*Factor III. Current implementation of curricular adjustments.*							
FIII.25. I adapt the practical activities to SEN	104.06	104.89	95.76	99.09	152.38	10.5	0.033
*Factor IV. Relationships and participation of students with disabilities.*							
FIV.27. Relationships between SEN students and their classmates are good.	84.06	102.16	124.72	85.5	117.15	10.72	0.030

* N None = 18; N 1–5 = 123; N 6–10 = 45; N 11–15 = 11; N more than 15 = 13.
